# SnS_2_-TiO_2_ Heterojunction Designed for Reductive Degradation of Contaminants of Emerging Concern

**DOI:** 10.3390/nano15130969

**Published:** 2025-06-22

**Authors:** Suresh Kumar Pandey, Sandra Romac, Josipa Papac Zjačić, Marijana Kraljić Roković, Marin Kovačić, Hrvoje Kušić, Boštjan Žener, Boštjan Genorio, Urška Lavrenčič Štangar, Ana Lončarić Božić

**Affiliations:** 1 Faculty of Chemical Engineering and Technology, University of Zagreb, Trg Marka Marulića 19, HR-10000 Zagreb, Croatia; skpandey@fkit.unizg.hr (S.K.P.); sromac@fkit.unizg.hr (S.R.); jpapac@fkit.unizg.hr (J.P.Z.); mkralj@fkit.unizg.hr (M.K.R.); mkovacic@fkit.unizg.hr (M.K.); abozic@fkit.unizg.hr (A.L.B.); 2 Department for Packaging, Recycling and Environmental Protection, University North, Trg dr. Žarka Dolinara 1, HR-48000 Koprivnica, Croatia; 3 Faculty of Chemistry and Chemical Technology, University of Ljubljana, Večna pot 113, SI-1000 Ljubljana, Slovenia; bostjan.zener@fkkt.uni-lj.si (B.Ž.); bostjan.genorio@fkkt.uni-lj.si (B.G.); urska.lavrencic.stangar@fkkt.uni-lj.si (U.L.Š.)

**Keywords:** SnS_2_-TiO_2_ composite, solar photocatalysis, contaminants of emerging concern, reductive degradation

## Abstract

Contaminants of emerging concern (CECs), including pharmaceuticals and perfluorinated compounds, pose a growing threat to water quality due to their persistence and resistance to conventional treatment methods. In this context, photocatalytic processes capable of promoting both oxidative and reductive transformations have attracted increasing attention. This study explores the synthesis and performance of a SnS_2_-TiO_2_ heterojunction photocatalyst, designed to facilitate such reactions under solar and UV-A light. The composite was synthesized via the hydrothermal method and thoroughly characterized for its morphological, structural, surface, and semiconducting properties. The results confirmed the formation of a type-II heterojunction with improved visible-light absorption and suppressed charge recombination. Photoelectrochemical measurements indicated enhanced charge separation and favorable band-edge alignment for reductive processes. Photocatalytic experiments with amoxicillin (AMX) and perfluorooctanoic acid (PFOA) revealed distinct degradation behaviors: AMX was predominantly degraded via superoxide-mediated reductive pathways, whereas PFOA exhibited limited transformation, likely proceeding via a combination of oxidative and reductive mechanisms. While overall removal efficiencies were moderate, this study highlights the role of band structure engineering and heterojunction design in tailoring photocatalytic behavior. The SnS_2_-TiO_2_ system serves as a promising platform for further development of composite materials to address the challenge of CECs in water treatment.

## 1. Introduction

As global freshwater resources face increasing pressure from climate change, population growth, and industrialization, water reuse has emerged as a vital strategy for ensuring a sustainable water supply [1]. However, the growing presence of contaminants of emerging concern (CECs) in treated wastewater poses a serious challenge to this effort. CECs are pollutants that, while present in the environment, remain largely unmonitored and unregulated. Many of them persist and bioaccumulate in the food chain, posing long-term ecological and health risks. Their complex chemical structures complicate detection, and conventional treatments often fail to fully remove them, resulting in their presence in drinking water [2]. Among the largest CEC groups are pharmaceuticals, which are widely used and persistent in aquatic environments. In recent years, poly- and perfluoroalkyl substances (PFASs) have also emerged as a major concern, drawing increased attention from both regulatory agencies and the scientific community [3]. Due to their unique structure, PFASs can resist harsh conditions and repel both oil and water [4,5]. These properties have driven their widespread use in the industry and consumer products, but also made them highly persistent in the environment. For example, perfluorooctanoic acid (PFOA), a representative PFAS, has been found in surface water, blood serum, and human breast milk [6,7,8,9]. Toxicological studies show that PFOA exposure can cause reproductive and developmental effects and liver damage and may increase the cancer risk [10,11,12]. Given their persistence and potential health impacts, developing efficient and cost-effective methods to remove PFASs from water is essential [13].

Various technologies have been developed and applied for CEC removal, including separation and degradation methods. Separation methods like activated carbon, ion exchange, and reverse osmosis have showed good efficiency but they produce secondary waste requiring further treatment. In contrast, emerging degradation technologies such as advanced oxidation/reduction processes (AO/RPs) aim for complete CECs mineralization [14,15]. AO/RPs employing reactive oxygen species (ROS), such as hydroxyl and sulfate radicals, can effectively break C–C bonds and detach head groups; however, PFASs can only be partially degraded due to an inability to cleave the highly stable carbon–fluorine (C–F) bonds. On the other hand, AO/RPs generating reductive species such as superoxide radicals and/or hydrated electrons—e.g., sonolysis, photocatalysis, radiolysis, and electrochemical processes—may directly target and break C–F bonds [16], thus presenting effective tools for PFAS degradation.

Among the available options, photocatalysis has showed significant potential, offering the degradation of CECs, including pharmaceuticals [17] and PFASs [15] as well, via both oxidative and reductive pathways. The latter involves the cleavage of heteroatoms, including defluorination under mild conditions as the main pathway for complete PFAS degradation [15]. Some pharmaceuticals are more susceptible to reductive than oxidative degradation [18], emphasizing the need for photocatalysts with optimal conduction band-edge (CBE) potentials [19]. Furthermore, if the band gap is suitable, the process can be activated under solar irradiation, offering a sustainable and energy-efficient solution [20]. Optoelectronic properties of photocatalysts can be tailored via metal or non-metal doping and construction of heterojunctions [21]. The choice of photocatalyst material is critical, as it influences light absorption, redox capability, and charge carrier dynamics. Titanium dioxide (TiO₂) is widely studied for its chemical stability, low cost, and strong redox abilities, but its wide band gap (~3.2 eV) restricts activity to UV light [22,23]. In contrast, tin disulfide (SnS₂), with a narrower band gap (∼2.1–2.3 eV) [24], responds to visible light, making it promising for solar-driven photocatalysis. In addition to its visible-light activity, SnS₂ offers several advantages, including non-toxicity, resistance to photocorrosion, and a negative conduction band potential (−0.52 eV), which is favorable for reductive reactions [19,25]. However, its narrow band gap also causes rapid electron–hole (*e*^−^/*h*^+^) recombination, reducing the efficiency of the overall treatment. To address such limitations, heterojunction photocatalysts have been developed to enhance light absorption, promote charge separation, and prolong charge carrier lifetimes [22,24,26]. Previous studies have demonstrated that combining SnS_2_ with TiO_2_ can enhance photocatalytic efficiency due to the formation of heterojunctions, which facilitates the separation of photogenerated charge carriers [27,28,29,30,31]. However, complete separation of *e*^−^/*h*^+^ pairs has not yet been achieved in TiO_2_/SnS_2_ composites. Therefore, there is significant potential to further improve the photocatalytic performance of TiO_2_/SnS_2_ systems [24].

This study investigates the photocatalytic degradation of selected CECs using a SnS_2_-TiO_2_ composite. PFOA, one of the most frequently detected PFASs in natural waters [32], and amoxicillin (AMX), selected due to its reported susceptibility to reductive rather than oxidative degradation [18], were used as model contaminants. The composite was synthesized, thoroughly characterized for its morphological, structural, surface, and optoelectronic properties, and tested under UV-A and simulated solar light to evaluate its efficiency in selected CECs’ degradation.

## 2. Materials and Methods

*Chemicals*: Titanium (IV) n-butoxide (Ti(OCH_2_CH_2_CH_2_CH_3_)_4_, Across Organics, USA), tin chloride pentahydrate (SnCl_4_×5H_2_O, Sigma Aldrich, St. Louis, MO, USA), thioacetamide (C_2_H_5_NS, Sigma Aldrich, St. Louis, MO, USA), glacial acetic acid (CH_3_COOH, Fluka, Seelze, Germany), and ethanol (C_2_H_5_OH, 96%, Carlo Erba Reagenti, France) were used for the photocatalysts’ synthesis. For the photoelectrochemical (PEC) tests, sodium sulfate (Na_2_SO_4_, Lachner, Neratovice, Czech Republic) was utilized as an electrolyte. Perfluorooctanoic acid (PFOA, C_7_F_15_COOH, Across Organics, Geel, Belgium) and amoxicillin (AMX, C_16_H_19_N_3_O_5_S, Thermo Scientific, Waltham, MA, USA) were used as CECs’ representatives. Ammonium acetate (CH_3_COONH_4_, LC-MS grade), formic acid (HCOOH, LC-MS grade), acetonitrile (CH_3_CN, HPLC grade), and water (H_2_O, HPLC grade) were provided by VWR Chemicals (Vienna, Austria) and were used along with methanol (CH_3_COOH, HPLC grade, Fisher Scientific, Brussels, Belgium) in the monitoring of the concentrations of selected CEC_S_ via LC/MS-MS. All the chemicals were used as obtained from the company without any further purification. Milli-Q water was used throughout the synthesis and degradation experiments.

*Synthesis of SnS_2_*: In order to synthesize SnS_2_, appropriate amounts of SnCl_4_×5H_2_O (0.2 M) and C_2_H_5_NS (0.4 M) were dissolved in a mixed solution of ethanol and glacial acetic acid (95:5 *v*/*v*) with stirring. After 15 min of stirring, the sample solution was transferred into a Teflon-lined autoclave further placed in an oven at 180 °C for 12 h. Thereafter, the cooled sample was centrifuged and washed several times with water and ethanol before drying at 65 °C for 16h.

*Synthesis of TiO_2_*: In a 100 mL solution of ethanol and glacial acetic acid (95:5 *v*/*v*), a 5 mL aliquot of Ti(OCH_2_CH_2_CH_2_CH_3_)_4_ was added under vigorous stirring. The resulting clear solution was transferred to an autoclave and sealed tightly. The autoclave was then kept in an oven and maintained at 180 °C for 12 h. The obtained white suspension was subjected to centrifugation and washed thoroughly to remove any impurities. The washed solid was then dried in an oven at 65 °C.

*Synthesis of SnS_2_-TiO_2_*: To prepare SnS_2_-TiO_2_ composites, the appropriate amounts of precursors used for the synthesis of SnS_2_ and TiO_2_ were mixed in a total volume of 100 mL of ethanol and acetic acid in a 19:1 *v*/*v* ratio. The mixture was stirred continuously for 15 min to ensure homogeneity. The resulting clear solution was transferred into a tightly sealed autoclave, which was then placed in an oven maintained at 180 °C for 12 h. After cooling, the sample was centrifuged to separate the solid material, which was thoroughly washed to remove any impurities. The washed solid was dried in an oven at 65 °C, and the resulting yellow material was finely ground using a mortar and pestle.

*Characterization*: The crystal structures of the samples were analyzed by X-ray diffractograms (XRDs) acquired by using a Rigaku Miniflex 600 (Tokyo, Japan) instrument with a Cu Kα target at 40 kV and 10 mA. A scanning electron microscope (SEM, Ultra Plus, Zeiss, Jena, Germany) equipped with energy dispersive X-ray spectroscopy (EDS) was used to determine the morphology and elemental composition of the material. The specific surface area and pore size distributions were measured on a Gemini 2380 Brunauer–Emmett–Teller (BET, Micrometrics, Norcross, GA, USA) analyzer. X-ray photoelectron spectroscopy (XPS) measurement was carried out on a Versa Probe 3 AD (PHI, Chanhassen, MN, USA) equipped with a monochromatic Al K*α* X-ray source to determine the elemental states present in the samples The surface charge properties of the prepared samples and mean particle size were analyzed by a Zetasizer (Malvern, UK) instrument; prior to measurement, a 0.2 mg/mL sample in water was sonicated at 25 °C for 5 min in an ultrasonic bath to ensure a uniform dispersion of the particles. The UV–visible diffuse reflectance spectroscopy (UV-DRS, Shimadzu, Kyoto, Japan) technique was used to determine the optical properties of the samples. The transfer behavior of photogenerated excitons was elucidated by recording the photoluminescence (PL) spectra of the photocatalysts on a fluorescence spectrophotometer (Varian Cary Eclipse, CA, USA). The photocurrent response and electrochemical impedance spectroscopy (EIS) measurements were performed using a standard three-electrode configuration, where a platinum (Pt) wire served as the counter electrode, and a saturated calomel electrode (SCE) was used as the reference electrode. The working electrodes were prepared by immobilizing the photocatalyst onto an FTO glass substrate. A 0.5 M Na_2_SO_4_ solution was used as the electrolyte. All PEC measurements were taken using a potentiostat/galvanostat (SP-150, Biologic, France) and a white LED was employed as the irradiation source for the electrodes; corresponding absorption spectra can be found in our previous study [32].

*Photocatalytic degradation procedure*: Photocatalytic degradation of PFOA and AMX were carried out in a custom-made, closed-batch, quartz-cover photoreactor, described in detail in our previous study [33]. The reactor has cooling water circulated through the jacket to maintain a constant temperature of the reaction system, while a magnetic stirrer (at 500 rpm) was used to homogenize the reaction solution, which was placed under the solar simulator (Oriel Newport, Irvin, CA, USA), equipped with a collimator, light source, 450 W Xe lamp (Osram, Munich, Germany), and air mass filter (AM 1.5 G) for simulating solar light. In experiments with UV-A light irradiation only, a UVB/C Blocking Filter along with an FSR-BG3 Colored Glass Bandpass Filter, used to narrow irradiation to the UVA region only (with maximum at 356 nm), were applied instead of an air mass filter. The closed reactor system was used to purge O_2_ and to create an inert atmosphere via N_2_ purging within the reactor system, thus preventing superoxide radical generation (O_2_^•−^). For the experiment, the reactor was filled with a 90 mL aqueous PFOA (12 μM) or AMX (50 μM) solution in which 1 g/L of photocatalyst was added. Prior to illumination, the suspension was stirred in the dark for 30 min to achieve adsorption–desorption equilibrium. During the experiment, the suspension was exposed to light (Solar or UV-A), and 0.5 mL samples were withdrawn at regular intervals (−30, 0, 10, 20, 30, 40, 50, 60, 75, 90). Each sample was filtered using a 0.45 μm cellulose filter (CA, Chromafil, Macherey-Nagel, Dueren, Germany) and taken for further analysis.

*Analytical methods*: The concentration of PFOA was monitored using ultra-high-performance liquid chromatography coupled with a triple-quadrupole mass spectrometer (LCMS/MS-8045, Shimadzu, Japan). The chromatographic separation was achieved on a Shim-pack C18 column (2.1 I.D. × 150 mm, Shimadzu, Japan). The mobile phase consisted of a binary mixture of phase A (2 mM ammonium acetate in 2% methanol) and phase B (2 mM ammonium acetate in 98% methanol), delivered at a flow rate of 0.25 mL/min. The gradient program was as follows: initially, 90% A and 10% B; after 4 min, ramped up to 20% A and 80% B for the next 4 min; then to 5% A and 95% B for 8 min, and finally, returned to the initial conditions and equilibrated for 3 min. The total runtime for each sample was 20 min. Tandem mass analysis was performed in multiple reaction monitoring (MRM) mode for enhanced specificity and sensitivity. For the detection of PFOA, the following transitions were utilized: *m*/*z* 169 and *m*/*z* 219 were selected as the qualitative ions, while *m*/*z* 369 was used as the quantification ion to minimize potential mass interference. To monitor the concentration of AMX, we utilized an HPLC (Series 20, Shimadzu, Japan) equipped with a UV-PDA detector (SPD-M20AVP, Shimadzu) using a reversed-phase C18 column (250 mm × 4.6 mm, 5 μm, Macherey-Nagel Nucleosil, Duren, Germany). Isocratic elution was carried out with a mobile phase consisting of 90% aqueous 50 mM formic acid and 10% acetonitrile at an overall flow of 1 mL/min. The oven temperature was 40 °C and the injection volume of analyte was 100 μL. AMX was detected at a wavelength of 273 nm.

## 3. Results

**Figure 1:**
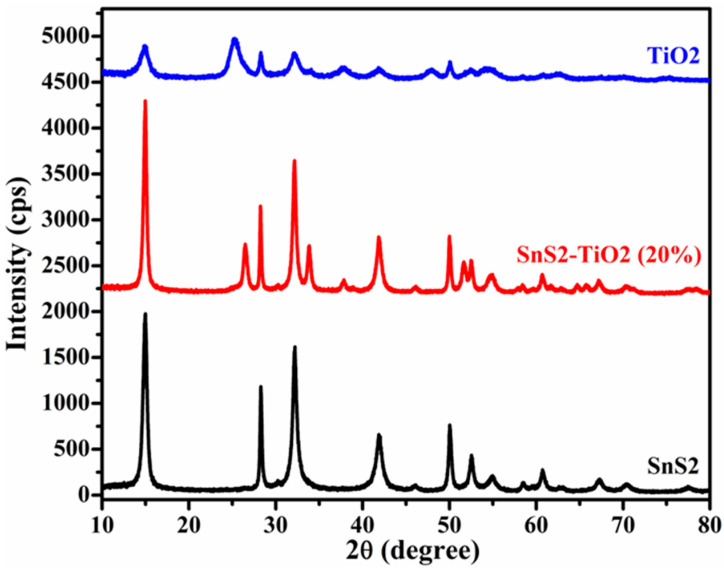
XRD patterns of SnS_2_, SnS_2_-TiO_2_ (20%), and TiO_2_.

In order to determine the crystal structure and phase purity, synthetized materials were characterized using XRD. The XRD patterns of pure TiO_2_, SnS_2_, and SnS_2_-TiO_2_ in combination are portrayed in Figure 1. The diffraction peaks of pure TiO_2_ and SnS_2_ are indexed well with the standard patterns of tetragonal (anatase; COD card no. 9015929) and hexagonal (berndtite; COD card no. 1011330) phases, respectively. In the case of the SnS_2_-TiO_2_ composite, the presence of characteristic peaks corresponding to both SnS_2_ and TiO_2_ confirms the successful formation of the SnS_2_-TiO_2_ heterostructure.

**Figure 2:**
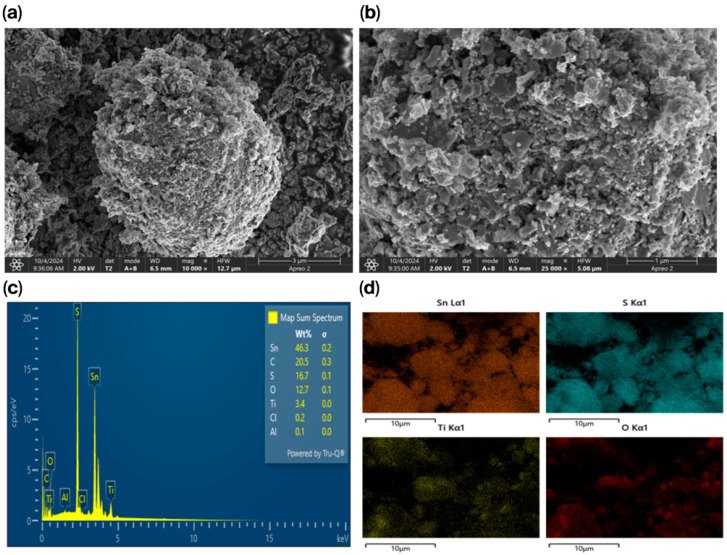
(**a**,**b**) SEM images of the SnS_2_-TiO_2_ composite, (**c**) EDS spectra of SnS_2_-TiO_2_, and (**d**) elemental mapping of SnS_2_-TiO_2_.

The SnS_2_-TiO_2_ composite was then submitted to morphological analysis. The SEM results are shown in Figure 2a,b at magnifications of 3 μm and 1 μm, respectively. The images reveal a rough surface with a flake-like morphology, which may be beneficial for adsorption due to the increased number of active sites provided by the rough texture [34]. The EDS data presented in Figure 2c confirms the presence of all the constituent elements of the SnS_2_-TiO_2_ composite, namely, Sn, S, Ti, and O, as expected. Additionally, the elemental mapping in Figure 2d demonstrates a homogeneous distribution of Ti and O over the SnS_2_ surface.

**Figure 3:**
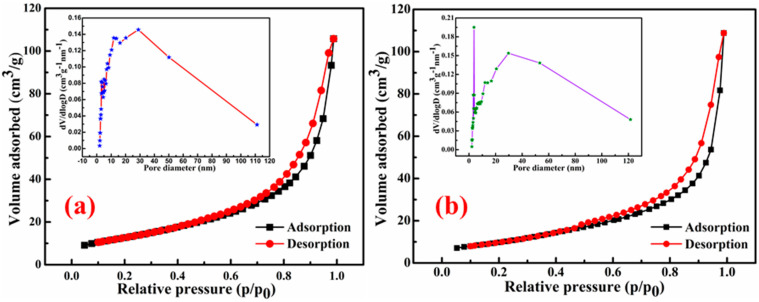
N_2_ adsorption–desorption isotherm and BJH plot (inserts) of (**a**) SnS_2_-TiO_2_ (20%) and (**b**) SnS_2_.

The specific surface areas (SSAs) of the studied materials, SnS_2_-TiO_2_ composite and sole SnS_2_, were determined using nitrogen (N_2_) adsorption–desorption measurements. Figure 3a presents the N_2_ adsorption–desorption isotherms for the SnS_2_-TiO_2_ composite. The SSA for the composite is calculated to be 46 m^2^/g, which is higher than that of pure SnS_2_ (37.19 m^2^/g), as shown in Figure 3b. This enhancement in surface area is likely attributed to the synergistic interaction between SnS_2_ and TiO_2_, leading to a more porous structure and increased surface exposure for the composite material. However, it should be pointed out that the SSA of the SnS_2_-TiO_2_ composite is smaller than that of pure TiO_2_ anatase (117 m^2^/g), synthetized in a previous study [35], as well as almost twice as small as that of commercial TiO_2_ P25 (90 m^2^/g) [36]. In addition, the SSA of the SnS_2_-TiO_2_ synthesized here with a ratio of SnS_2_ and TiO_2_ components of 80% to 20% is much smaller than that of the composite composed of the same components but with a different ratio of SnS_2_ (27.5%) and 72.5 (TiO_2_), for which it yielded a 160 m^2^/g SSA [28]. The clear reason is a larger content of the SnS_2_ component, which has a smaller SSA comparing to TiO_2_. A hysteresis loop indicates that it is a type IV isotherm, which suggests a mesoporous structure of the SnS_2_-TiO_2_ composite. The pore size diameter of the composite ranges from 2 to 20 nm, as can be observed from the Barrett–Joyner–Halenda (BJH) plot (inserts of Figure 3a).

**Figure 4:**
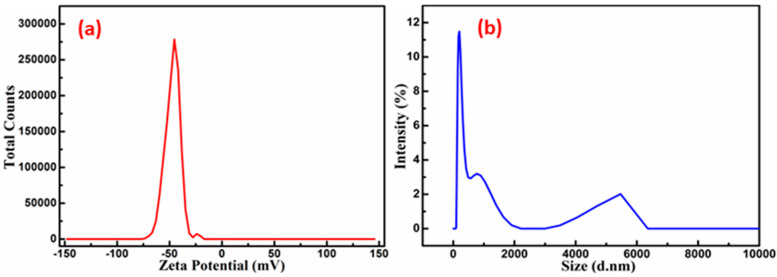
(**a**) Zeta potential and (**b**) particle size distribution of SnS_2_-TiO_2_ (20%).

The zeta potential is a key characteristic of particles, influencing both their stability and overall properties. A more pronounced zeta potential, whether positive or negative, typically enhances the stability of particle suspensions due to the electrostatic repulsion between particles carrying the same charge, which prevents aggregation. In the current study, the zeta potential was measured at −45.19 mV, as shown in Figure 4a. The negative zeta potential value of the SnS_2_-TiO_2_ composite suggests an effective dispersion and stability of the particles in suspension. The average particle size of the composite measured by dynamic light scattering (DLS) techniques is 100 nm (Figure 4b), with a solution polydispersity index (PDI) of 0.35.

**Figure 5:**
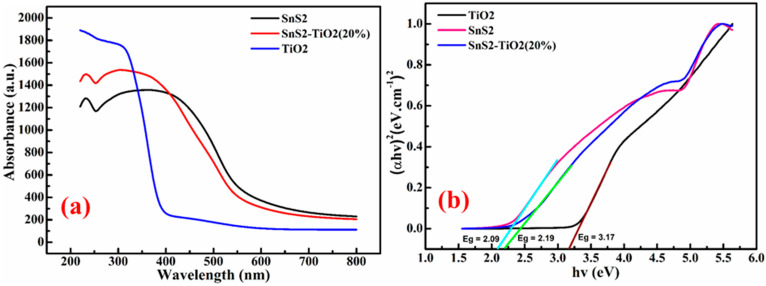
(**a**) UV-Vis absorbance spectra and (**b**) Tauc plot of TiO_2_, SnS_2_, and SnS_2_-TiO_2_ (20%) samples.

Determining the band gaps of semiconducting materials is essential for a proper understanding of their photocatalytic mechanisms. The absorbance data of the samples (Figure 5a), obtained using a UV-DRS instrument, were used to construct the Tauc plots for SnS_2_, TiO_2_, and SnS_2_-TiO_2_. The Tauc plots, displayed in Figure 5b, were derived using the following relation:

$${\left(\alpha h\vartheta \right)}^{2}=\left(h\vartheta -E_{g}\right)$$

where *α* represents the absorption coefficient, *h* is Planck’s constant, *ν* is the frequency of light, and *E*_g_ is the optical band gap energy. The optical band gap energies, as calculated from the above equation, were found to be 2.09 eV for SnS_2_, 3.17 eV for TiO_2_, and 2.19 eV for the SnS_2_-TiO_2_ heterostructure. The DRS results indicate that both SnS_2_ and the SnS_2_-TiO_2_ composite exhibit strong absorption in the visible-light region, while TiO_2_ primarily absorbs in the UV region.

**Figure 6:**
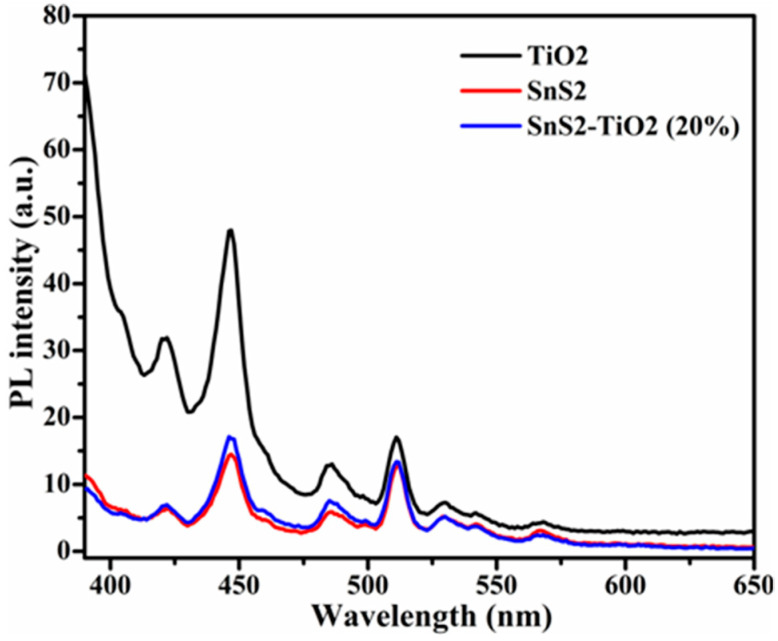
PL spectra of SnS_2_, TiO_2_, and SnS_2_-TiO_2_ (20%).

Photoluminescence is a valuable technique for evaluating the recombination rate of photogenerated excitons (*e*^−^/*h*^+^ pairs). The PL emission intensity is directly correlated with the recombination of electrons and holes; therefore, a lower PL intensity signifies a restrained recombination rate and also effective charge separation [37,38]. In this study, a wavelength of 375 nm was used as the excitation source to record the PL spectra of the samples. The results presented in Figure 6 display that the PL intensity of the SnS_2_-TiO_2_ composite is very close to that of pure SnS_2_, and that both lie below pure TiO_2_. This behavior can be attributed to the formation of a heterostructure with SnS_2_ as the dominant component, where the difference in band-edge positions facilitates the recombination of electrons from the TiO_2_ conduction band (CB) with holes from the valence band (VB) of SnS_2_, which is typical for n-n heterojunctions [39]. As a result, the remaining photogenerated charges, electrons in CB of SnS_2_ and holes in VB of TiO_2_, are effectively separated and stable, and the overall recombination probability of excitons is reduced. However, such assumptions need to be proven via photoelectrochemical (PEC) tests.

**Figure 7:**
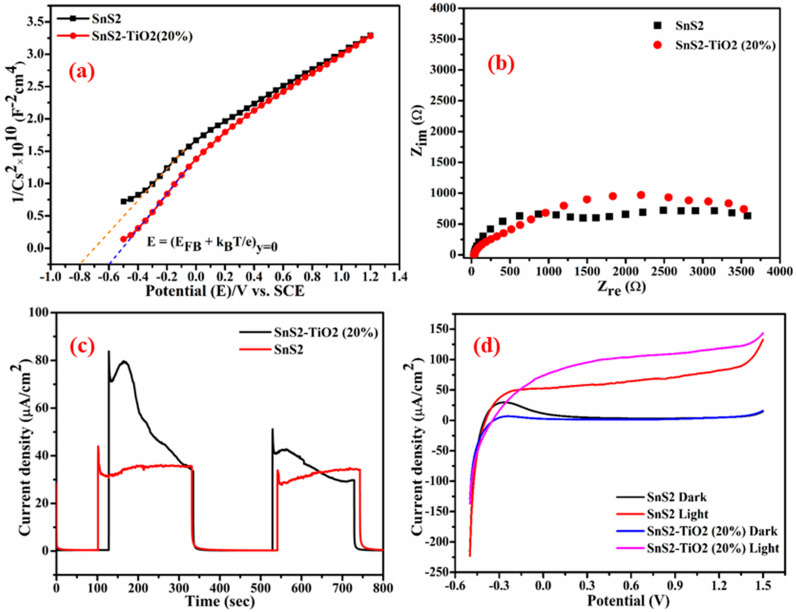
(**a**) Mott–Schottky curves, (**b**) EIS Nyquist plots, (**c**) transient photocurrent response, and (**d**) linear sweep voltammograms of SnS_2_ and SnS_2_-TiO_2_ (80:20) electrodes in 0.5 M Na_2_SO_4_.

The flat band potentials (*E*_FB_) at the semiconductor/electrolyte interface were determined from Mott–Schottky plots recorded under dark conditions using Equation [40].

$$\frac{1}{C^{2}}=\frac{2}{{\varepsilon \varepsilon }_{0}A^{2}eN_{D}}\left(E-E_{FB}-\frac{k_{B}T}{e}\right)$$

where *C* and *A* are the interfacial capacitance and area, *ε* is the dielectric constant of the semiconductor, *ε*_0_ is the permittivity of free space, *N*_D_ is the number of electron donors, *E* is the applied voltage, *k*_B_ is Boltzmann’s constant, *T* is the absolute temperature, and *e* is the electronic charge. From the above relation, both *E*_FB_ and *N*_D_ can be extracted, taking into account that values for *ε* and *A* are known [41,42]. The Mott–Schottky plots for the synthesized samples are presented in Figure 7a. The positive slopes of the Mott–Schottky plots indicate that both SnS_2_ and the SnS_2_-TiO_2_ composite are n-type semiconductors [40], in which electrons are major charge carriers. It was previously reported by our group that TiO_2_ is an n-type semiconductor, with a flat band potential of −0.29 V [35]. The calculated flat band potentials for SnS_2_ and the SnS_2_-TiO_2_ composite are found to be −0.577 V and −0.377 V vs. SHE, respectively. It can be seen that all investigated photocatalytic materials have CB positions at more negative potentials than 0 V vs. SHE, which is usually important for hydrogen generation via photocatalytic reactions, but also it indicates that this material may have the capacity to reduce PFASs. Materials with a CB positioned more negatively than 0 V can provide the necessary thermodynamic driving force to reduce both protons (for hydrogen generation) and PFASs. Specifically, studies have shown that photocatalysts like TiO_2_ with suitable band alignment can transfer electrons to PFOA, facilitating its stepwise reduction and degradation into less harmful products [43]. However, successful PFOA degradation also depends on the generation of reactive species like superoxide (O_2_^●−^) and the material’s overall catalytic properties, not just the conduction band position.

Open circuit potential (OCP) measurements provide insight into the photoactivity of the material, helping us to understand its behavior in photocatalytic applications for the degradation of organic pollutants. The OCP under illumination provides information regarding the photoactivity of the composite, as shifts in OCP are correlated with photoinduced charge separation. The response values obtained by OCP measurement, charge recombination rate (k_r_), and photovoltage (ΔE) of composite and pure components are summarized in Table 1, calculated based on the equations presented in Figure S1 (Supplementary Materials), which we used in our previous study, where more detailed explanations of performed OCP and calculations are provided [33].

It can be noted that the composite has the highest photovoltage and lowest recombination rate, which is likely to have a positive effect on the photocatalytic degradation of selected CECs (PFOA and AMX). This is in accordance with the previously reported data [35], where a composite was also prepared from TiO_2_ and SnS_2_, but with a much smaller amount of SnS_2_.

Nyquist plots for the composite and SnS_2_ are shown in Figure 7b. For the dark condition, a higher impedance value was registered for composite SnS_2_-TiO_2_ (80:20) material compared to pure SnS_2_ (Figure S2, Supplementary Materials). For the illuminated electrode, although a different response was obtained, indicating different properties of space charge layer and different charge transfer dynamics [44], the difference was not significant.

Chronoamperometry was employed to investigate the charge separation efficiency and current stability over time under continuous light illumination. A constant potential of 0.5 V was applied under dark and chopped LED light (Figure 7c). As shown in Figure 7c, no current response was observed in the dark, while illumination produced a photocurrent due to the separation of photogenerated *e*^−^/*h*^+^ pairs. The composite electrode showed a higher photocurrent than pure SnS_2_, indicating improved charge separation and photocatalytic activity. However, the composite’s photocurrent gradually declined over time, suggesting possible photocatalyst degradation. In contrast, SnS_2_ exhibited a more stable, though lower photocurrent. The enhanced performance of the composite is attributed to the formation of a type II heterojunction between SnS_2_ and TiO_2_, promoting efficient charge transfer. The synergistic effects of the two components (TiO_2_ aiding charge separation and SnS_2_ enhancing light absorption) are critical to the observed photoelectrochemical behavior.

The results of an LSV experiment (Figure 7d) indicate that the dark current response is close to zero while the response of the illuminated electrode is anodic current. The photocurrent (*I*_ph_) is the result of the photoactivity of a material, and its value is the difference between light and dark current responses (*I*_ph_ = *I*_light_ − *I*_dark_). Therefore, the good PEC activity of the investigated material is evident in Figure 7d, as *I*_light_ >> *I*_dark_. In addition, from the obtained results, it is evident that the composite resulted in higher currents compared to SnS_2_. However, it is less stable, which is evident from the current decrease with each cycle (Figure S3, Supplementary Materials).

**Figure 8:**
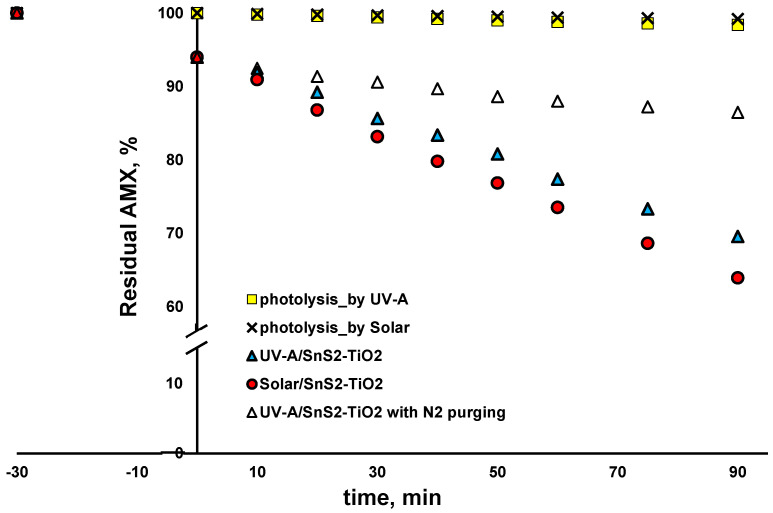
Degradation profiles of AMX using SnS_2_-TiO_2_ composite under different irradiation sources (UV-A and solar) (conditions: natural pH (5.5), γ (catalyst dose) = 1 g/L (where applicable)).

The activity of synthetized SnS_2_-TiO_2_ (80:20) toward selected CECs, AMX and PFOA, was examined under solar and UV-A irradiation. As can be seen from Figure 8, the blank tests with direct solar and UV-A photolysis (no catalysts) yielded negligible changes in AMX concentration (<1 and <2% removal within 90 min). AMX was adsorbed at SnS_2_-TiO_2_ during a dark period in the amount of 6%, which remained stable with the prolonged time when an additional 90 min was tested in blank experiments, where irradiation was not applied. Accordingly, it can be concluded that an adsorption equilibrium was achieved during the 30 min dark period applied in the photocatalytic experiment (Figure 8), and that all removed AMX after exposing the reaction solution to either solar or UV-A irradiation was due to degradation. As can be seen from Figure 8, somewhat higher AMX removal (36.1%) after 90 min was recorded when solar irradiation was applied, compared to the case with UV-A light (30.4% of AMX removed). Our previous study clearly exhibited that AMX is mainly degraded by a reductive mechanism by the UV-A/TiO_2_ system [18], i.e., either by *e*^−^, which may be valid for the amount adsorbed on the surface of the catalyst only, or O_2_^•−^, which is responsible for bulk reactions [45,46]. For that reason, AMX was selected as a CEC undergoing reductive degradation. In order to test that assumption, we conducted an additional experiment, where the reaction vessel was purged with N_2_ in order to remove O_2_, thus preventing O_2_^•−^ formation. As can be seen from Figure 8, only 13.5% of AMX was removed after 90 min. Considering there was 6% adsorption during the dark period, it can be concluded that AMX was degraded only up to 7.5% in this experiment. That result clearly shows that AMX mainly undergoes reductive degradation, and mainly via O_2_^•−^ in bulk. The reason for the somewhat higher degradation rate of AMX under solar compared to under UV-A light may be the higher recombination rate under light of a higher energy (UV-A), which presumably left less charge available for the generation of ROS (primarily O_2_^•−^).

**Figure 9:**
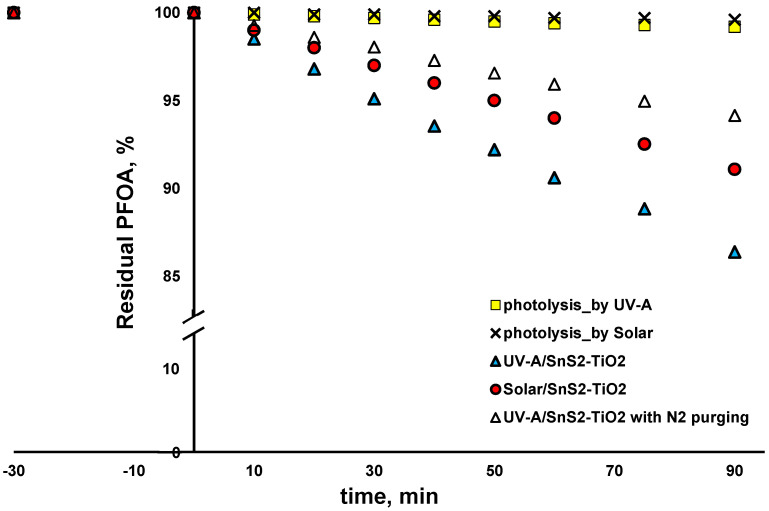
Degradation profiles of PFOA using SnS_2_-TiO_2_ composite under different irradiation sources (UV-A and solar) (conditions: natural pH (5.5), γ (catalyst dose) = 1 g/L (where applicable)).

The same set of experiments was performed with another selected CEC, PFOA, which also undergoes reductive degradation via the cleavage of C-F bonds, thus leading to mineralization. However, PFOA may also undergo oxidation as well, which would result with shorter-chain PFASs, but not mineralization [15,47,48]. As can be seen from Figure 9, PFOA was only negligibly degraded by direct solar and UV-A photolysis (<0.5%), speaking in favor of its recalcitrance in the environment. It should be also noted that PFOA was not adsorbed on the SnS_2_-TiO_2_ composite during the dark period. Such behavior indicates that PFOA would not be subject to direct photocatalytic mechanism produced by photogenerated charges (*e*^−^ and *h*^+^). Accordingly, only bulk degradation via the generated ROS, hydroxyl radicals (HO^•^) and O_2_^•−^, can be expected. As can be seen, solar/SnS_2_-TiO_2_ yielded slightly lower degradation comparing to the case using UV-A irradiation after 90 min treatment (8.9 < 13.6%, respectively). Such results are the opposite of those in the case of AMX, where solar-driven photocatalytic treatment yielded somewhat higher CEC degradation. The plausible explanation can be found in the fact that, most probably, AMX mainly undergoes reductive treatment (as proven in Figure 8), while in the case of PFOA, the oxidative pathway yielding shorter-chain PFASs may come forth as well [48]. Under UV-A light, TiO_2_ can be activated as well, yielding more ROS, primarily HO^•^ responsible for the oxidative degradation of PFOA, than under solar light. Hence, in spite of a somewhat higher recombination rate, as mentioned above for the case of AMX under UV-A light, such effect may be overcome by generating HO^•^ more effectively, which would yield higher PFOA degradation under UV-A than under solar. In order to investigate the portions of PFOA degraded via the oxidative and reductive pathways, we conducted an additional experiment in a N_2_ atmosphere, as in the case of AMX above. As PFOA did not adsorb onto SnS_2_-TiO_2_, direct degradation via photogenerated charge carriers would not occur, while an inert atmosphere would prevent the generation of O_2_^•−^, thus we recorded PFOA degradation in such an experiment, amounting to 5.8% (Figure 9), which can be assigned to the HO• pathway. Accordingly, it can be concluded that the contributions of HO• and O_2_^•−^ to PFOA degradation by UV-A/SnS_2_-TiO_2_ are similar, amounting to 43% and 57%, respectively.

**Figure 10:**
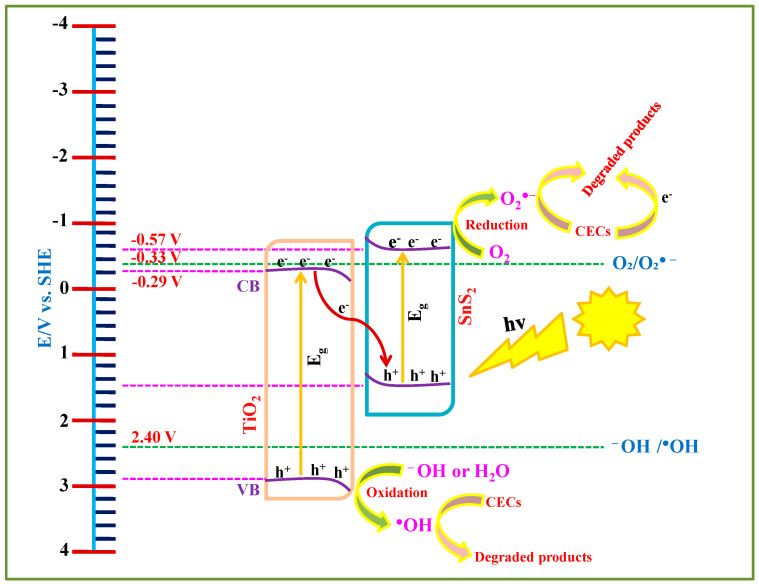
Plausible schematic representation of the photocatalytic pathway.

Based on the DRS, PL, and PEC analyses, a schematic representation of the overall photocatalytic process is presented in Figure 10. The edge positions of the CB and VB of the materials were calculated using flat-band potential values obtained by Mott–Schottky and band gap values via DRS analysis. Based on these calculations, the CB edges of TiO_2_ and SnS_2_ are located at −0.29 V and −0.577 V, while the VB edges are positioned at 2.88 V and 1.51 V, respectively. Upon irradiating with suitable light sources (solar and UVA, in our case), electrons are excited to their CB while leaving behind corresponding holes in the VB. However, the photogenerated holes in the VB of SnS_2_ and electrons in the CB of TiO_2_ are not thermodynamically favorable for producing HO^•^ (E_OH_^−^_/OH_^•^ = 2.40 V) and O_2_^•−^ (E_O2/O2_^•−^ = −0.33 V), respectively. As a result, these charge carriers tend to remain inactive and are likely to recombine under the influence of internal electric fields and band bending effects at the interface. In contrast, the electrons in the CB of SnS_2_ and holes in the VB of TiO_2_ possess sufficient redox potential and remain available for photocatalytic activity. These active charge carriers contribute to the degradation of selected CECs, either directly through electrons/holes or via generating O_2_^•−^ and HO^•^. However, as stated above, PFOA did not adsorb onto the SnS_2_-TiO_2_ composite (Figure 9), thus the involvement of photogenerated charge carriers (electrons and holes) in its direct degradation can be neglected, and accordingly, only bulk degradation over formed ROS is possible in the case of PFOA.

**Figure 11:**
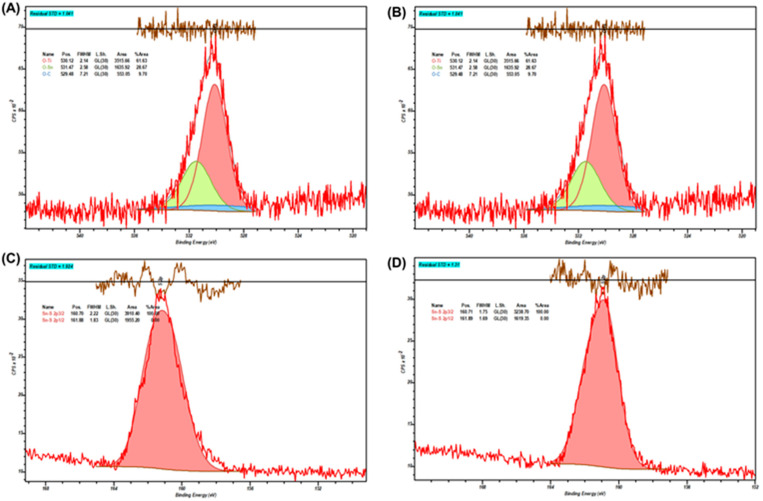
Deconvoluted high-resolution X-ray photoemission spectra (HRXPS) for O 1s in the pristine SnS_2_-TiO_2_ (**A**) and post-photocatalysis (**B**); S 2p3/2 spectra for the pristine SnS_2_-TiO_2_ photocatalysis (**C**) and post-photocatalysis (**D**).

In order to test changes on the SnS_2_-TiO_2_ composite surface, which may imply its stability, XPS analysis of photocatalysts prior to and after the treatment was performed; the results are presented in Figure 11. In Table 2, the binding energies (BEs) for the O 1s and S 2p3/2 regions of SnS_2_-TiO_2_ are listed. After photocatalysis treatment, a slight decrease in the binding energy of the Ti-O bond can be observed. Such a shift indicates a subtle reduction in the titanium centers, i.e., a partial conversion of Ti^4+^ to Ti^3+^ or the formation of oxygen vacancies in the TiO_2_ lattice. Such an effect can be attributed to the migration of the photogenerated electrons from SnS_2_ to TiO_2_. On the other hand, the O-Sn bond BE has risen considerably. This higher binding energy implies the formation of surface hydroxyl groups on SnS_2_ [49]. In turn, this could imply the interactions of HO^•^ formed on TiO_2_ or water with the surface exposed to the Sn centers in SnS_2_. However, the binding energy of the S 2p3/2 peak remains virtually unchanged, i.e., no oxidation of S^2−^ in the bulk or on the surface is evident, implying a good stability of the composite.

## 4. Conclusions

This study presents a SnS_2_-TiO_2_ (80:20) heterojunction composite as a photocatalyst for the degradation of selected CECs, specifically AMX and PFOA. Structural characterization via XRD confirmed the coexistence of anatase TiO_2_ and berndtite SnS_2_ phases, with SEM revealing a flake-like morphology and EDS confirming a uniform elemental distribution. Nitrogen adsorption–desorption measurements showed a moderately enhanced specific surface area for the composite (46 m^2^/g) compared to pure SnS_2_ (37.2 m^2^/g).

Optical measurements revealed visible-light absorption (band gap of 2.19 eV), while the composite’s intermediate PL intensity, relative to the pure components, suggests a partial suppression of charge recombination via heterojunction-facilitated electron transfer from SnS_2_ to TiO_2_. Additionally, photoelectrochemical characterization indicated a relatively high light response (ΔE = 212.6 mV) and a low recombination rate (*k*_r_ = 0.0068 s⁻^1^), consistent with more efficient charge separation. Electrochemical analyses confirmed that the SnS_2_-TiO_2_ composite behaves as an n-type semiconductor, while the positions of valence and conduction bands enable the effective generation of both HO^•^ and O_2_^•−^. Despite moderate photocurrent stability issues, the composite exhibited an improved charge separation efficiency compared to SnS_2_ alone.

Photocatalytic experiments showed the measurable degradation of AMX (36.1% under solar irradiation, 30.4% under UV-A), with the results indicating a predominantly reductive degradation pathway mediated by superoxide radicals. PFOA degradation was more limited (up to 13.6% under UV-A) and proceeded via a combination of oxidative and reductive mechanisms, suggesting that complete mineralization remains challenging under the tested conditions. XPS analysis before and after treatment indicated only minor changes in surface chemistry, supporting the structural stability of the composite during photocatalysis.

Overall, while the SnS_2_-TiO_2_ heterojunction achieved moderate removal efficiencies, it demonstrated promising attributes in terms of band alignment, charge transport, and photocatalytic response under solar and UV-A irradiation. Further optimization of the SnS_2_-TiO_2_ ratio, structural robustness, and operating conditions may enhance its applicability for water treatment targeting CECs.

## Figures and Tables

**Table 1:** Summarized parameters obtained by the OCP technique for the as-prepared materials.

Material	Photovoltage (ΔE, mV)	Charge Recombination Rate (*k*_r_, 1/Sec)
TiO_2_ (HT)	54.19	0.030 [35]
SnS_2_ (HT)	166.33	0.0132
SnS_2_-TiO_2_(20%)	212.56	0.0068

**Table 2:** High-resolution X-ray photoemission spectroscopy (XPS) spectra for O 1s and S 2p3/2 in pristine SnS_2_-TiO_2_ and post-photocatalytic experiments, along with the difference in binding energy (ΔBE) between the two.

XPS Region and Bond	Peak Position, eV		
	Pristine	Post-Photocatalysis	ΔBE, eV
O 1s, O-Ti	530.29	530.12	−0.17
O 1s, O-Sn	530.43	531.47	1.04
S 2p3/2, Sn-S	160.70	160.71	0.01

## Data Availability

The datasets collected and analyzed in this work are available from the corresponding authors upon reasonable written request.

## Supplementary Materials

The following supporting information can be downloaded at https://www.mdpi.com/article/10.3390/nano15130969/s1, Figure S1. Results of OCP measurements. Figure S2. Nyquist plots of SnS_2_ and SnS_2_-TiO_2_ (80:20) in dark. Figure S3. LSV in different cycles of SnS2 (a) and SnS2-TiO2 (80:20) (b).
