# Supplementary material for: SnS2-TiO2 Heterojunction Designed for Reductive Degradation of Contaminants of Emerging Concern
Source: Nanomaterials (Basel). 2025 Jun 22;15(13):969. doi: 10.3390/nano15130969 (PMC12251373; doi:10.3390/nano15130969)
Supplement: Supplementary file 1 [file nanomaterials-15-00969-s001.zip › nanomaterials-3673890-supplementary.pdf]

Supplementary Materials

SnS_2-TiO_2 heterojunction designed for reductive degradation of contaminants of emerging concern

Suresh Kumar Pandey[1], Sandra Romac[1], Josipa Papac Zjačić[1], Marijana Kraljić Roković[1], Marin Kovačić[1], Hrvoje Kušić[1,2,*], Boštjan Žener[3], Boštjan Genorio[3], Urška Lavrenčič Štangar[3], Ana Lončarić Božić[1]

[1] University of Zagreb, Faculty of Chemical Engineering and Technology, Trg Marka Marulića 19, HR-10000 Zagreb, Croatia; skpandey@fkit.unizg.hr (S.K.P.); sromac@fkit.unizg.hr (S.R.); jpapac@fkit.unizg.hr (J.P.Z.); mkralj@fkit.unizg.hr (M.K.R.); mkovacic@fkit.unizg.hr (M.K.); abozic@fkit.unizg.hr (A.L.B.)

[2] University North, University center Koprivnica, Trg dr. Žarka Dolinara 1, HR-48000 Koprivnica, Croatia;

[3] University of Ljubljana, Faculty of Chemistry and Chemical Technology, Večna pot 113, SI-1000 Ljubljana, Slovenia; Bostjan.Zener@fkkt.uni-lj.si (B.Ž.); Bostjan.Genorio@fkkt.uni-lj.si (B.G.); Urska.Lavrencic.Stangar@fkkt.uni-lj.si (U.L.Š.)

* Correspondence: hkusic@fkit.unizg.hr (H.K.); Tel.: +385 1 4597 160

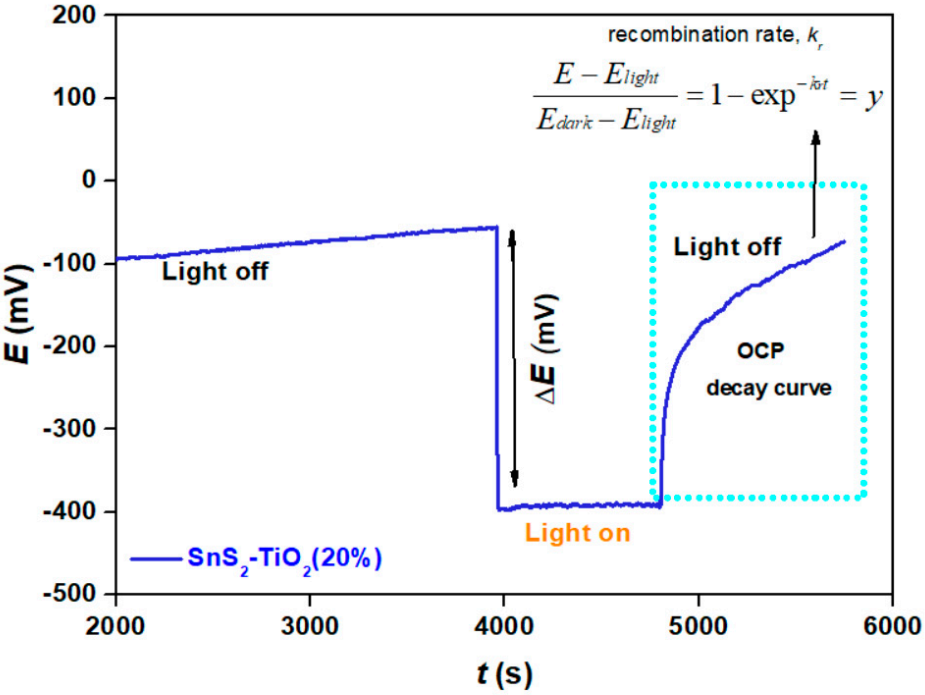

Figure S1. Results of OCP measurements.

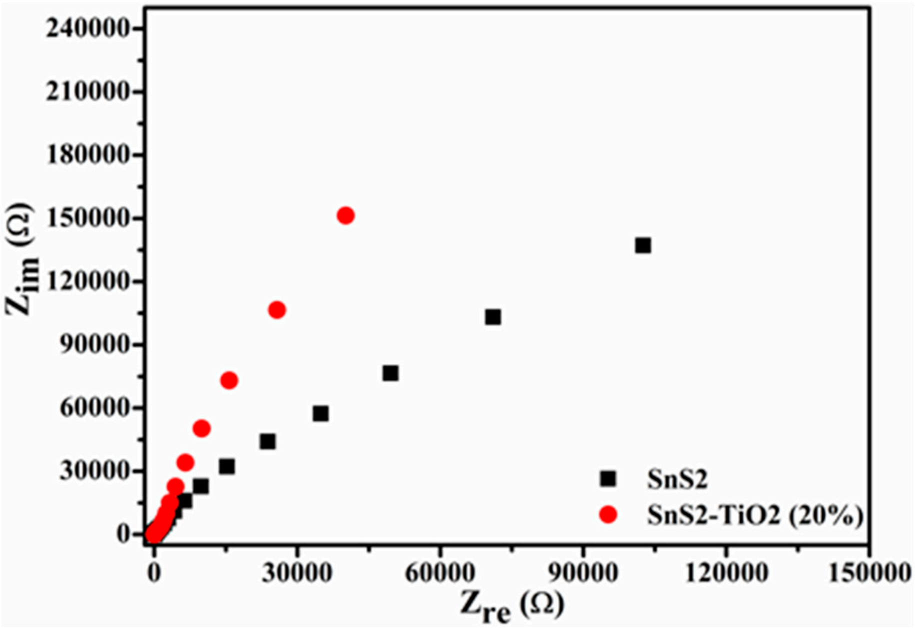

Figure S2. Nyquist plots of SnS_2 and SnS_2-TiO_2 (80:20) in the dark.

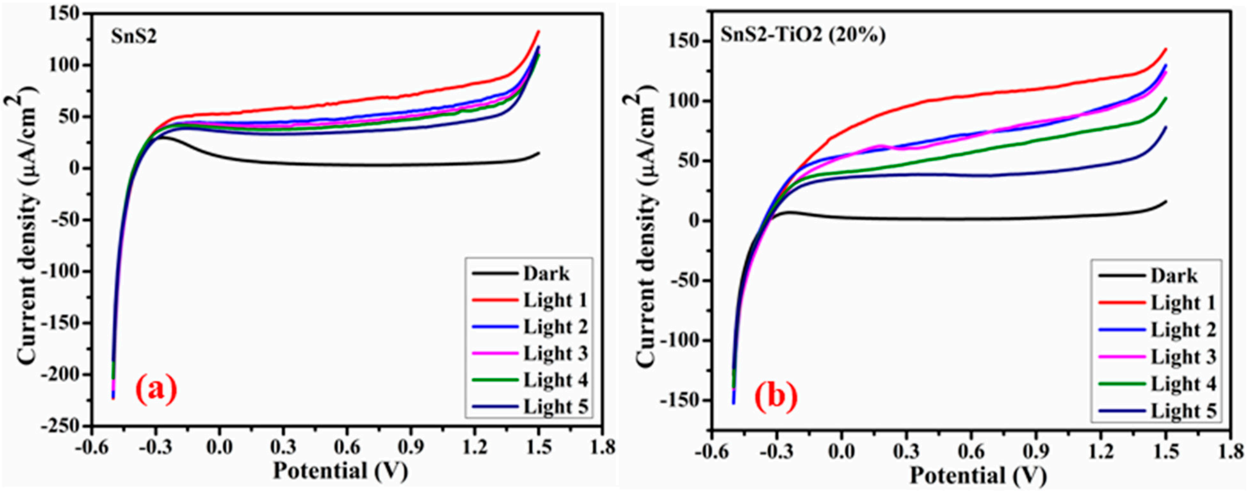

Figure S3. LSV in different cycles of SnS_2 (**a**) and SnS_2-TiO_2 (80:20) (**b**).
